# Fabrication of high-quality glass microfluidic devices for bioanalytical and space flight applications

**DOI:** 10.1016/j.mex.2020.101043

**Published:** 2020-08-27

**Authors:** Matin Golozar, Wai K. Chu, Laura D. Casto, Jeremy McCauley, Anna L. Butterworth, Richard A. Mathies

**Affiliations:** a Department of Chemistry and Biophysics Graduate Group, University of California, Berkeley, CA 94720, United States; b Space Sciences Laboratory, University of California, 7 Gauss Way, Berkeley, CA 94720, United States; c Department of Biology, Massachusetts Institute of Technology, Cambridge, MA 02139, United States

**Keywords:** Capillary electrophoresis, Glass microfabrication, Lab-on-a-chip, Astrobiology, Biosignature detection

## Abstract

Microfabricated glass microfluidic and Capillary Electrophoresis (CE) devices have been utilized in a wide variety of applications over the past thirty years. At the Berkeley Space Sciences Laboratory, we are working to further expand this technology by developing analytical instruments to chemically explore our solar system. This effort requires improving the quality and reliability of glass microfabrication through quality control procedures at every stage of design and manufacture. This manuscript provides detailed information on microfabrication technology for the production of high-quality glass microfluidic chips in compliance with industrial standards and space flight instrumentation quality control.

• The methodological protocol provided in this paper includes the scope of each step of the manufacturing process, materials and technologies recommended and the specific challenges that often confront the process developer.
• Types and sources of fabrication error at every stage have been identified and their solutions have been proposed and verified.
• We present robust and rigorous manufacturing and quality control procedures that will assist other researchers in achieving the highest possible quality glass microdevices using the latest apparatus in a routine and reliable fashion.

## Graphical abstract

**Figure:**
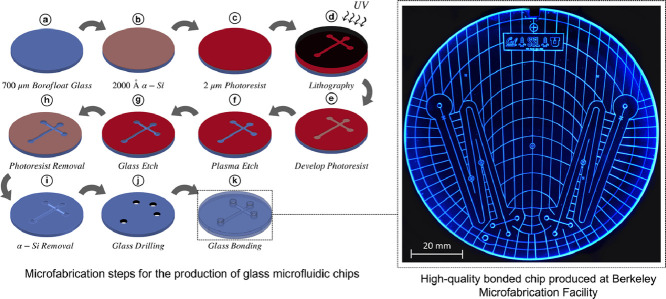

**Table:** Specifications Table

Subject Area	Chemistry
More specific subject area	*Bioanalytical Chemistry, Astrobiology*
Method name	Fabrication technology for the production of high-quality glass microfluidic chips
Name and reference of original method	• P.C. Simpson, A.T. Woolley, R.A. Mathies, Microfabrication Technology for the Production of Capillary Array Electrophoresis Chips, Biomed. Microdevices. 1 (1998) 7–26 • C.A. Emrich, H. Tian, I.L. Medintz, R.A. Mathies, Microfabricated 384-lane capillary array electrophoresis bioanalyzer for ultrahigh-throughput genetic analysis, Anal. Chem. 74 (2002) 5076–5083
Resource availability	• https://nanolab.berkeley.edu/ • https://bnc.qb3.berkeley.edu/ • https://openwetware.org/wiki/Protocols

## Method details

### Background

Since the introduction of microfabricated total analysis systems (µTAS) in 1990 [1], there has been an explosion of interest in utilizing microfluidics and lab-on-a-chip technologies for a broad range of applications in the life and chemical sciences [2,3]. As the applications of microfluidics have expanded, numerous materials including silicon, quartz/fused silica, glass, ceramics, hydrogels, polymers, and papers have been used for developing these devices bearing diverse functionalities [2]. Among these materials, glass has remained the preferred material for capillary electrophoresis (CE) in a miniaturized planar format [3,4]. Glass wafers are advantageous because their surface properties are very similar to those of conventional fused-silica capillaries. Glass substrates provide a robust platform in which to fabricate high-quality micron precision CE channels, reliably exhibiting higher separation efficiencies and faster analysis times compared to other materials. Furthermore, glass is a natural choice for bioanalysis since glass is inert to most chemicals and biochemicals and does not adsorb reagents or chemicals like soft PDMS lithographic devices. Finally, glass is optically transparent with very low-fluorescence background so when coupled with laser-induced fluorescence (LIF), high sensitivity detection of targeted analytes can be realized [4]. However, glass fabrication is more demanding than many soft fabrication techniques leading to the temptation to cut corners in the fabrication process thereby producing low-quality glass devices. The purpose of this paper is to restate proper glass fabrication techniques using the latest available apparatus and procedures to achieve the highest possible quality devices.

Three decades have passed since CE systems were first integrated on a planar glass chip using photolithography techniques [5] and used to separate a mixture of fluorescent dyes [6] and later to separate fluorescently labeled amino acids [7], [8], [9]. Since then, much more advanced and complex CE devices have been fabricated from glass and further integrated into lab-on-a-chip analytical systems to perform high-speed and high-resolution electrophoretic separation for an expanding range of applications. Key work includes devices for DNA fragment separations [10], DNA sequencing [11,12], PCR product amplification and analysis [13], clinical diagnostics and pathogen detection [14,15], cell sensors [16] and forensic identification [17].

Microfluidic chemical analysis systems are also being developed for the chemical exploration of our solar system [4,[18], [19], [20], [21]. At the UC Berkeley Space Sciences Laboratory (SSL), we are focused on building fluorescence detection-based in situ microfluidic instruments to look for organic biosignatures in our solar system that are indicative of past or present life at Enceladus (Saturn) and Europa (Jupiter). The Enceladus Organic Analyzer (EOA) is being developed to probe the ice plumes that jet out from Enceladus [4] while the Microfluidic Organic Analyzer for Biosignatures (MOAB) will explore the icy surface of Europa (http://eoa.ssl.berkeley.edu/). Both instruments are being engineered in a flight-capable configuration and share a common core analysis engine that combines a microfabricated glass wafer for high resolution CE separations, a programmable microfluidic analyzer (PMA) for sample processing, and a fluorescence detection system for high sensitivity LIF detection and quantitation [22].

In order to obtain high levels of sensitivity and resolution for fluorescence analysis in capillary electrophoresis, excellent quality glass CE wafers need to be designed and fabricated. The Mathies group pioneered the development of robust manufacturing methods producing high-density CE devices with high-quality micron size channels [12,22]. To enable this technology for space flight applications, we were interested in improving the quality and reliability of glass fabrication capabilities to industrial standards.

**Fig. 1:**
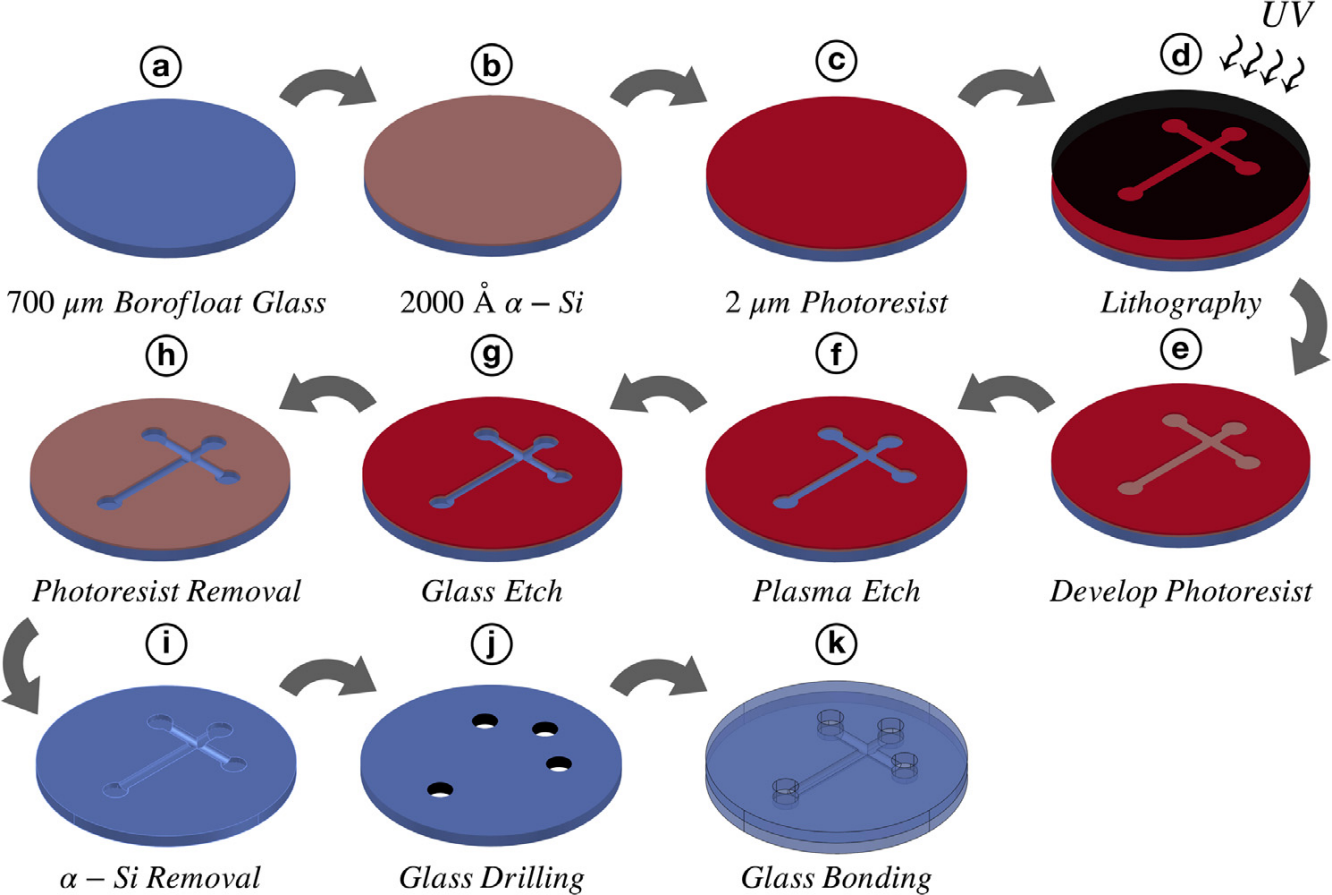
Outline of steps in the fabrication process of glass capillary electrophoresis chips: (a) A 700 µm thick blank borofloat glass wafer; (b) Deposition of 2000 Å a-Si in a LPCVD furnace; (c) Spin coating of a 2 µm layer of positive photoresist; (d) Standard UV lithography; (e) Photoresist development; (f) Plasma etch of a-Si; (g) Wet etch of glass using 49% HF; (h) Photoresist removal; (i) a-Si removal; (j) CE reservoir drilling into a 1.1 mm blank borofloat glass wafer; (k) Thermal bonding of etched and drilled wafers together to form CE channels.

In overview, the fabrication of glass CE wafers is performed using standard photolithography and glass wet microchannel etching techniques as summarized in Fig. 1. Borofloat glass wafers (100 mm dia., 0.7 mm thick) are first coated with a 2000 Å amorphous silicon (a-Si) sacrificial layer using a low-pressure chemical vapor deposition (LPCVD) furnace. Next, the wafer is spin coated with positive photoresist (S1818, Shipley) to get the desired thickness of 2 µm, followed by soft baking to semi-harden the photoresist. The microchannels are then patterned photolithographically by exposure to 436-nm UV light through a contact aligner with a chrome-photomask. The exposed photoresist is developed and removed using a 1:1 solution of MICROPOSIT developer concentrate and water. The pattern is further transferred into the a-Si layer by SF_6_ plasma etch, thus creating a hard mask suitable for glass etching. CE channels are etched isotropically 30 µm deep using hydrofluoric acid (49%). The remaining photoresist and a-Si is stripped from the wafer using acetone and SF_6_ plasma, respectively. CE reservoirs are drilled using 1.5 mm diameter diamond bits on a CNC mill into a separate blank borofloat glass wafer (100 mm dia., 1.1 mm thick). Finally, etched and drilled wafers are thoroughly cleaned and thermally bonded together at 668°C for 6 h to make a finished device.

In this manuscript, we provide detailed information on the entire glass CE wafer fabrication process, the scope of each step, materials and technologies recommended and the specific challenges that often confront the process developer. In addition, types and sources of fabrication errors at every stage have been identified and their solutions have been proposed and verified. The methodological protocol provided in this paper is in sufficient detail to enable other researchers to replicate the entire methodology and to microfabricate high-quality and defect-free glass wafers with dense CE separation channels in a routine and reliable fashion.

## Materials and methods

### Materials

Precise thickness Schott borofloat glass wafers: thickness ± 0.025 mm (polish on one side with scratch/dig better than 60/40 from Alpha Precision Inc, IL, USA). Normal Schott borofloat glass backing wafers: thickness ± 0.1 mm (Precision Glass and Optics, CA, USA). Ceramic Macor blocks (Technical Products Inc, WI, USA). Powdered Graphite Lubricant. Texwipe TechniCloth wipers. Diamond drill bits. Pine resin. Vinyl Surface Protection Tapes (Texas Technologies, Inc). NovaClean detergent. Piranha (mixture of sulfuric acid H_2_SO_4_, water, and hydrogen peroxide H_2_O_2_). Acetone (C_3_H_6_O). Isopropyl alcohol (IPA – C_3_H_8_O). De-ionized water (DI water with a resistivity of ≈ 18 megaohm-cm). Positive photoresist (Shipley, S1818). Hexamethyl-disilazane (HMDS). MICROPOSIT developer. Hydrofluoric acid (10:1 49% HF).

### Methods

#### Wafer handling

Vacuum wands are the preferred way of picking and handling wafers and should be used to pick up the wafers on the back side and close to the edge. Manual tweezers are an alternative option for handling wafers. To transfer the wafers from one cassette to another, match up their pin and holes to mate the two cassettes and flip over to transfer from one into the other. Spin-rinse dry is the preferred way to dry wafers without touching them. If spin-rinse dry is not an option, wafers can be dried with a nitrogen (N_2_) gun. In order to do this, first blow nitrogen gas over the back side of the wafer and place it with its dried face down on a cleanroom wiper (Technicloth) and continue to blow-dry the front face until there are no more water residues on the surface and edges of the wafer.

#### Standard cleaning procedure

Borofloat glass has demonstrated the best etch quality among various types of glass being characterized as possible substrates for CE chips [23]. Borofloat is a borosilicate glass that produces flat glass in a float process without the need for any mechanical polishing. Borofloat glass wafers must be cleaned vigorously prior to LPCVD a-Si deposition to ensure both surfaces are free of any sort of contamination, such as grease, particles, dust, etc. The description of the standard wafer cleaning protocol has been previously documented by Chu and Mathies [24] and all steps should be carried out thoroughly on both sides of the wafers. In summary, wafers are initially rinsed with water and NovaClean liquid to remove grease and particulates, followed by rinsing wafers in an ultrasonic acetone bath with the etched side facing down. Note that glass wafers require polishing on one side to meet the desired thicknesses. This will form surface and sub-surface defects and strains on the polished/ground surface which will further develop and expand into a large groove in the etching process. Therefore, the other side (the superior side that is kept untouched and pristine) is the preferred surface of the wafer for CE manufacturing and needs to be distinguishable throughout the entire fabrication process. Next, the wafers are cleaned in piranha solution for 10 min at 80°C to remove organic residues, followed by quick-dump rinse (QDR) and spin-rinse-dry (SRD) for the final rinsing and spin-drying.

#### LPCVD preparation

The Tystar16 LPCVD (Low Pressure Chemical Vapor Deposition) furnace in Berkeley Microfabrication Facility was characterized and used to deposit 2000 Å a-Si on clean borofloat glass wafers. In LPCVD processes, the vacuum deposition chamber is heated to initiate a reaction of a process gas over the wafer surface, producing high quality thin film properties and uniformity. Cleanroom facilities usually have process monitoring data taken on a monthly basis on furnaces which provide statistical process control data such as deposition rate and film thickness uniformity. However, these data must be used as a starting point and a series of experiments need to be performed on test wafers (dummy wafers) to achieve target value film thickness prior to the deposition of actual wafers.

Tystar16 is a Non-MOS sub-atmospheric furnace designed for doped/undoped poly and amorphous polysilicon film deposition. This furnace has been manufactured by Tystar Corporation and is capable of processing 4” and 6” wafers. LPCVD systems deposit films on both sides of at least 25 wafers at a time. Wafers without scratches and chips must be selected and go through the Standard Cleaning Procedure prior to loading them into the furnace. The LPCVD process for a-Si deposition is described in detail elsewhere [24], but we will briefly restate the process parameters below to emphasize the most important steps and procedures.

#### LPCVD process and recipe

The process parameters to deposit 2000 Å a-Si are as follows:

• Deposition temperature: 525°C
• SiH_4_ flow rate: 100 SCCM
• PH_3_ flow rate: 0 SCCM
• Deposition pressure: 300 mTorr
• Deposition time: 2 h 50 min (must be adjusted for each furnace to achieve 2000 Å)

**Fig. 2:**
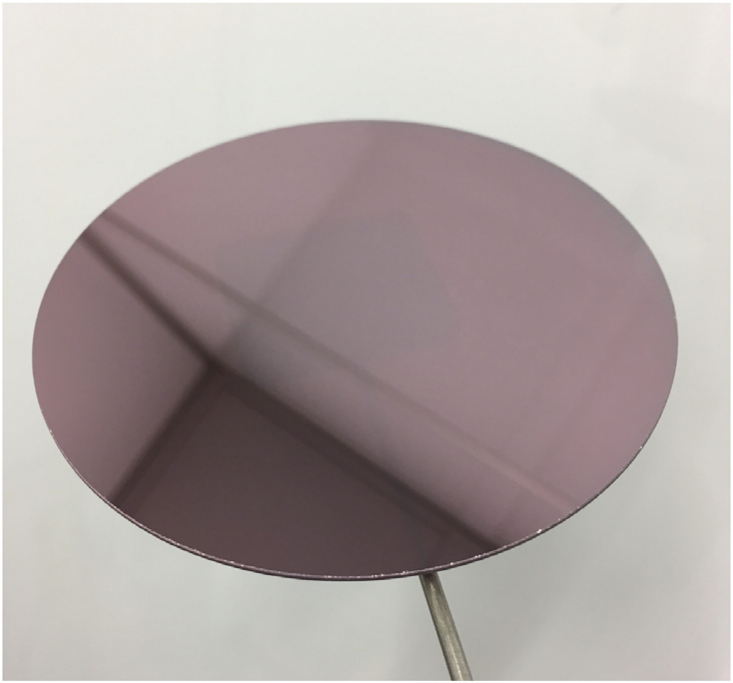
Image of a wafer with 2000 Å a-Si coating.

Silane (SiH_4_) is the source of the silicon deposited in the film and phosphine (PH_3_) must be used in case of phosphorous doping. Since we are interested in undoped a-Si deposition, the PH_3_ gas flow rate must be set to zero. As can be seen in Fig. 2, a successful a-Si coating, with the uniform thickness of approximately 2000 Å, will yield a deep reddish color. It is worth mentioning that thinner coatings (< 2000 Å) have a bluish color and are more likely to exhibit pinhole defects. Additionally, as the coating becomes thicker (> 2000 Å) the color shifts towards dark/black until it transitions into polysilicon which will negatively affect the etch quality of the glass substrate.

#### Photoresist spin coating preparation

There are several steps to take prior to the application of photoresist:

1. Remove particulates and dust from the substrates with N_2_ gas and select wafers that are free from scratches or smudges, especially if such defects would interfere with the CE channel locations. Defects on the glass surface or in the deposited a-Si layer could affect subsequent spin coating and etching processes.
2. Dehydration bake in the oven at 120°C for 30 min followed by dehydration bake on a hotplate at 200°C for 5 min.
3. Vapor HMDS priming of the wafers to promote photoresist adhesion and prevent its delamination during development and wet etch processes.
  • HMDS standard priming recipe: Total time of 11 min including 2 min of prime with no pump and purge cycles involved. HMDS coating following the standard recipe will remain on the wafer surfaces for about one hour until it disappears. Therefore, it is recommended to proceed with the photoresist coating immediately after the wafers are primed with HMDS.

#### Spin coating protocol

In our application, we use Shipley S1818 as a positive photoresist and spin it onto the wafers to define the etch pattern. The spin-coating and soft baking parameters for S1818, to get the desired film thickness of 2 µm, are listed below:

• Place the wafer on chuck at the center of the Photoresist Spinner with the superior surface face up.
• 500 rpm with the acceleration of 100 rpm/s to spread photoresist for 5 s in combination with 2500 rpm with the acceleration of 500 rpm/s to spin for 25 s (30 s total spin time). This will produce a film thickness of 2 µm.
• Apply photoresist during the first 5 s directly from the bottle onto the wafer so that about 90% of the glass surface is covered. It is not necessary to completely cover the surface as spinning will spread the photoresist over the entire surface and the HMDS primer will ensure that it adheres evenly. Pay attention not to introduce bubbles while pouring photoresist.
• Once the spin coating is finished, allow the wafer to rest on the chuck for 15–20 s so that the resist firms up, followed by transferring the wafer to the bake station for soft baking.

#### Soft baking protocol

• Transfer the wafer to a 120°C hot plate for 2.5 min to semi-harden the photoresist.
• Cool for 10-15 s on a cooling block.

#### Alignment and exposure

After the wafers have been coated with photoresist and soft baked, they are taken to the mask aligner for mask alignment and pattern exposure. The necessary exposure time should be set according to the particular type of photoresist and the power of the lamp being used. For our application, S1818 photoresist needs a minimum of 80 mJ/cm^2^ exposure. Therefore, 5 s exposure time was used for the measured lamp intensity of 16 mJ/cm^2^. Prior to loading the mask into the mask aligner, make sure that the chrome mask is free of photoresist residue stuck on the feature areas or particles that may be blocking UV illumination from reaching the photoresist. The N_2_ gun can be used to blow the particles off the mask. To remove photoresist residue off the mask, apply acetone followed by IPA using a swab with a lollipop tip to clean the affected area. Once the mask is cleaned, place it chrome side down on the mask vacuum chuck in a way that the chrome side touches the glass wafer during exposure. Alignment must be done with at least a 50 µm gap distance between the mask and the wafer to avoid scratching the surface of the photoresist while moving the wafer in x, y, and z positions for alignment adjustments. After the wafer is aligned, it is pushed up in contact with the mask before it is exposed to UV light. Once exposure is complete, transfer the exposed wafer to the chemical sink for photoresist development.

#### Photomask making

Photomask drawing is done using standard CAD software and sent to a company specialized in photomask printing (Front Range PhotoMask Inc, AZ, USA). Typical resolution for transparent film photomask is in the range between 50 and 10 µm. However, if higher resolution (in the range between 1 and 10 µm) is required, a photomask glass substrate with chrome is recommended. The mask lateral dimensions (x and y) are determined by the available exposure tool. In our fabrication, Karl Suss MA6 Mask Aligner (ksaligner) is used for fine lithography down to 1 micron and better. This aligner is capable of processing 4” and 6” wafers and can support 5” and 7” photomasks. Since we are using 100 mm diameter glass substrates and the minimum feature size on the mask is 5 µm, 5” square glass chrome masks are used.

**Fig. 3:**
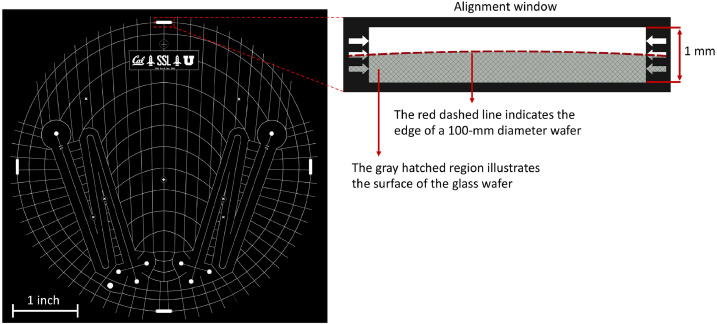
5” square glass chrome mask containing alignment features with open windows to observe the edge of the wafer during the alignment procedure. Black is chrome.

In order to ensure etched and drilled holes are aligned to one another during glass bonding, the mask design must include a set of alignment marks. As there are no alignment marks on the wafer prior to the lithography process, the mask must contain alignment features with large open windows for detecting the edge of the wafer from the front surface of the mask during alignment (Fig. 3). This is the most accurate and easiest way to assure that the wafer is perfectly centered within the mask substrate. As can be seen from the enlarged image in Fig. 3, arrows have been drawn to the sides of the alignment window, indicating expected locations of the edge of a 100 ± 0.5 mm diameter wafer. There are three arrows on each side to compensate for the tolerance of the diameter of the glass wafers which is typically ± 0.5 mm. Furthermore, the glass drilling that takes place on a separate blank substrate using a CNC mill, must be conducted by picking the center of the wafer as the reference point for drilling. By this procedure the etched and drilled holes will be aligned to one another during bonding.

#### Photoresist development

**Fig. 4:**
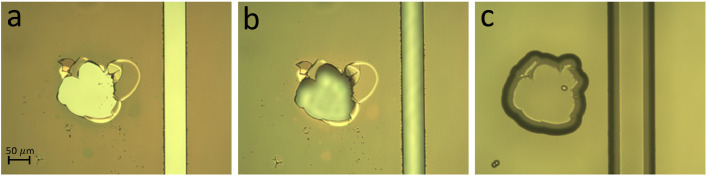
Progression of defects that are associated with the lithography process. Pattern failures originating from lithography process are further transferred to the silicon and glass layers causing defects in the channels. (a) Defect in the photoresist layer next to a channel feature; (b) Transfer of defect from photoresist to the silicon layer; (c) Transfer of defect from silicon to the glass layer.

For S1818 photoresist with a thickness of 2 µm, immerse each wafer in developer (1:1 solution of MICROPOSIT developer (MP DEV) concentrate and water) and gently agitate for 1 - 1.5 min. Once this development is finished, rinse each wafer with water, blow dry, and inspect the resist pattern under a microscope. As can be seen in Fig. 4a, the feature walls and exposed areas should be even and clearly defined. Any defect originated during the photolithography step will transfer to the silicon mask and subsequently to the glass layer when etched with HF (Fig. 4). Additional tips and tricks to overcome common failures during resist development can be found in our protocol site [25].

#### Wafer preparation for silicon etch

Silicon etch is done with an etchant gas (usually SF_6_) using reactive-ion etching (RIE). The plasma etching system used in our fabrication processes is called Plasma-Thermal PK-12 RIE (ptherm). Prior to loading the wafers into the etching system chamber and to prevent the Si on the back of the wafers from being etched off, it is recommended to put Vinyl Surface Protection Tapes on the back of the wafers. To do this, put down a layer of tape on a flat surface and place the wafer on top from its back. Use a razor blade to trim around the edges, flip the wafer over and squeeze the air bubbles out towards the edges of the wafer using the back of the blade or a hard-plastic card.

#### RIE silicon etch

If the RIE chamber appears to have contamination issues, it is recommended to perform oxygen plasma treatment prior to loading the wafers. To do so, run an O_2_ scourge for 5-10 min with 100% flow at 300 W power. After cleaning the chamber, load the wafers face up. Perform photoresist descum (ashing) by running O_2_ at 100% flow for 1 min at 100–150 W power. Photoresist descum is performed to clean the wafer surface for the subsequent RIE etching step by removing any resist residue left in the patterned regions. Such residual layers can interfere with the subsequent processes and affect the uniformity of the etching rate across the wafer. Immediately after the descum step, run SF_6_ gas at 80% flow for 1-1.5 min at 200 W to etch the Si hard mask layer. These times can be adjusted if necessary. For example, if a different photoresist is used as a masking layer, both ashing and etching steps should be adjusted to obtain required process uniformity. Once the Si etching process is complete, unload the wafers and carry them to the chemical sink for the glass etch process.

#### Glass etch

**Fig. 5:**
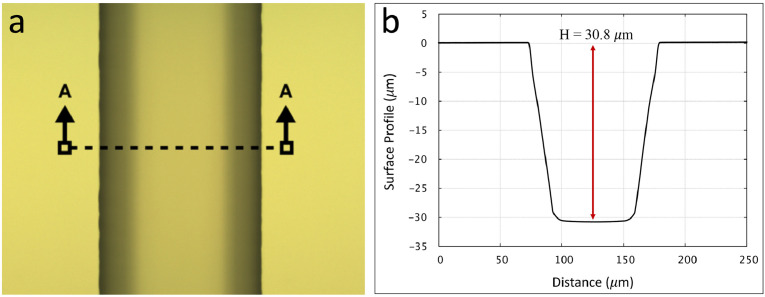
(a) Microchannel fabricated in borofloat glass etched to a depth of ≈ 30 µm using wet etching in a 49% HF and amorphous silicon as the etch mask; (b) Surface profile of the etched channel at the cross section of A-A along the microchannel width.

Before starting the glass etch process, ensure the back side of the wafer remains covered with the surface protection tape. The tape is chemically resistant to HF and will act as an extra protective layer to resist etching in case there are bare glass areas on the back side. Additionally, the etch rate of the material to be etched must be known. In our case, channels were wet etched 30 µm deep into the wafer (borofloat glass) using 49% HF with the etch rate of 6 µm/min (total etch time of 5 min). The etch rate can be determined by using a dummy glass wafer which is partially masked by photoresist. The wafer is locally dissolved in HF and the etch rate is determined by monitoring and measuring the recessed etched region using a profilometer. To start the etching process, fill a PTFE evaporating dish with enough 49% HF acid to entirely cover the wafers and gently agitate throughout the etch to obtain a desirable channel depth. Use plastic (Fluorocarbon - CTFE) tweezers that resist HF in order to handle wafers. Once the etching process is over, immediately transfer the wafer with plastic forceps to a separate beaker of water, rinse extensively with DI water, blow dry and use a surface profilometer to measure depth. Repeat the etch cycle as necessary until the desired etch depth is achieved as illustrated in Fig. 5. This figure presents a microchannel being etched into a borofloat glass to a depth of ≈ 30 µm with the surface profile of the channel at the cross section of A-A along its width. After the wet etch process is complete and the etched pattern is inspected under a microscope, it is time to remove the remaining photoresist from the mask followed by removing the a-Si hard mask from the glass substrate. Remove the surface protection tape from the back side of the wafer prior to proceeding to the next step.

#### Resist removal and mask clean

Acetone can be used to dissolve the remaining photoresist on the photomask. Immerse the wafer in a beaker filled with acetone and ensure that it is completely covered with the solvent. Rinse the acetone off the wafers with IPA and then DI water rinse to clean off IPA. Finally, repeat the RIE silicon etch step to completely remove the remaining a-Si coating from both sides of the wafer.

**Fig. 6:**
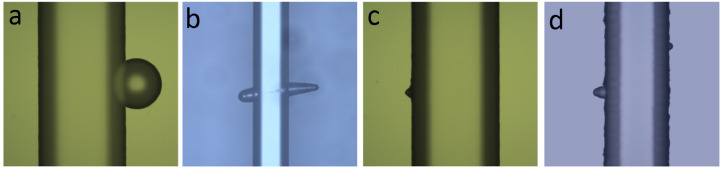
Optical images of defects caused by (a) a pinhole in the a-Si mask; (b) a scratch on the glass substrate; (c) contamination (dust/particle) prior to LPCVD; and (d) the presence of moisture in the a-Si layer.

A list of tips is included below in order to provide a troubleshooting guide for the most common failures during glass etch process. In capillary electrophoresis, even a tiny defect and imperfection in the capillary walls will distort the uniformity of the flat electroosmotic flow (EOF) profile and will therefore reduce the separation efficiency by broadening the injected plugs [26]. Using the following tips and techniques, high success rates (up to 90%) for fabrication of wet etched CE channels have been obtained. Fig. 6a illustrates a pinhole defect that is a characteristic defect present in all etch masks. This defect is due to the presence of inevitable dust and chips of metals in the evaporation chamber, where a-Si deposition takes place. Even though the photoresist covers the pinholes, wet etchants can easily penetrate through photoresist alone. Among various etch mask materials (such as chrome, gold/chrome, a-Silicon, etc.), a-Si has demonstrated excellent resistance to HF and the fewest defects [23]. Fig. 6a is a demonstration of a microchannel with a pinhole defect in its vicinity after the borofloat substrate has been etched with HF to a depth of 30 µm.

Fig. 6b shows an example of defects that are associated with microscratches on the surface of glass substrates. This is for the most part an inherent defect to the type of glass or the condition of the surface at the time of the etch. These scratches are due to polishing and sample handling errors during manufacturing and will further expand as the wafer is etched in HF and can damage chip functionality if one happens to cross any of the CE channels. Such defects can be eliminated through annealing the polished glass before processing or by pre-etching the glass wafers prior to depositing the a-Si protective etch mask [23].

Fig. 6c details the features that are usually observed due to the presence of dust or particles on the glass substrates that result from insufficient cleaning of the wafers prior to the etch mask deposition step. Finally, Fig. 6d indicates a case when the a-Si layer contains an excess of moisture prior to starting the photolithography process. The dehydration step before the application of photoresist prevents the occurrence of such defects.

#### Glass drilling

Depending on the range of hole diameters, aspect ratios and surface roughness, there are a wide variety of conventional and non-conventional drilling techniques [27] that can be used to drill glass wafers. In our fabrication, we use mechanical drilling and the CE reservoirs were drilled with 1.5 mm diameter diamond bits on CNC mill. Mechanical drilling is widely used as it is the most common and relatively low-cost method, enabling production of straight holes with minimal roughness and good surface quality in a short machining time. To prevent potential damage to the CE microchannels on the etched substrate during the rough drilling and hole preparation process, drilling must take place on a blank substrate before bonding. When mechanically drilling brittle materials, cracks and chips are generated at the exit surfaces. This can be prevented by placing another glass substrate underneath the blank wafer as a backing wafer during the drilling process. For this purpose, the blank wafer must be resin-bonded to a backing wafer on a hot plate at 225°C. Follow the resin bonding procedure described here [28] to obtain a uniform and bubble-free resin film between the two wafers.

Machining is done while the resin-bonded wafers are immersed in water or diamond drilling coolant solution to cool down the drill site and reduce the temperature gradient. Reservoirs are drilled with 1.5 mm diameter diamond drill bits on a CNC mill at a high speed of at least 10,000 rpm. When drilling with a high-speed drill press, the drill bits will wear faster and thus should be replaced with a fresh one approximately every 15 holes to maintain high quality drilled holes. After the holes have been drilled, the blank wafer must be separated from the backing wafer and cleaned following the cleaning procedure described in our protocol site [28].

#### Glass bonding

Prior to bonding the patterned and drilled wafers, they are taken back to the cleanroom for a thorough cleaning as described in the Standard Cleaning Procedure section. Immediately after cleaning, each pair of wafers should be direct-bonded face to face and placed into a transfer box for transport to the bonding station. Wafers are then prepared and bonded together at 668°C by thermal compressing sintering in an atmospheric furnace following the glass bonding procedure documented elsewhere [29]. Here we restate the bonding process to emphasize key points because this is a critical step where process developers often have difficulties.

1. Choose two polished flat Macor ceramic disks and pour a small amount of graphite powder onto their bonding surfaces.
2. Mate the two Macor disks with the graphite powder in between and use a circular motion to move one disk over another. This helps to evenly distribute the powder over the bonding surfaces. Continue this process until both surfaces receive a very thin and even coating (Fig. 7a).

**Fig. 7:**
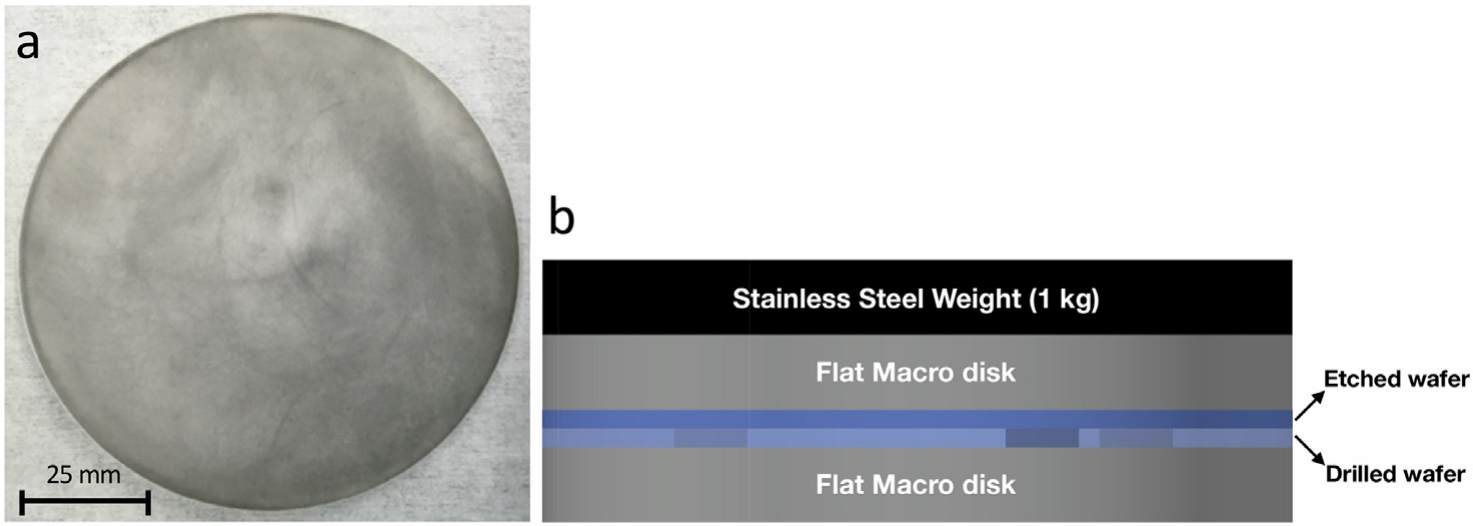
(a) A Macor ceramic disk with a thin, even coating of graphite powder on top; (b) Glass bonding schematic.
3. Take the mated glass wafer pair out of the transfer box carefully enduring that they do not shift relative to one another and sandwich them between the Macor disks with the drilled surface facing down.
4. Place a 1 kg stainless-steel weight on top of the Macor-wafer assembly.
5. Carefully transfer the entire assembly (Fig. 7b) into the atmospheric furnace.
6. Depending on your wafer size, choose the desired bonding process variables (ramp rates, temperature, time) and set the furnace to run your program.

Borofloat glass wafers are bonded at 668°C over the course of certain amount of time depending on the wafer size. Usual bonding parameters for 4” and 6” wafers are as follows:

• 4” wafers: 668°C for 6 h (ramping up temperature: 10 degrees per min)
• 6” wafers: 668°C for 8 h (ramping up temperature: 10 degrees per min)

Once the bonding process is finished and the wafers are cooled down, remove the chip and inspect the bond quality under microscope. Particularly search for voids that could cause a chip to be nonfunctional, such air pockets trapped in the bonding interface that intersect with CE microchannels or reservoirs.

**Fig. 8:**
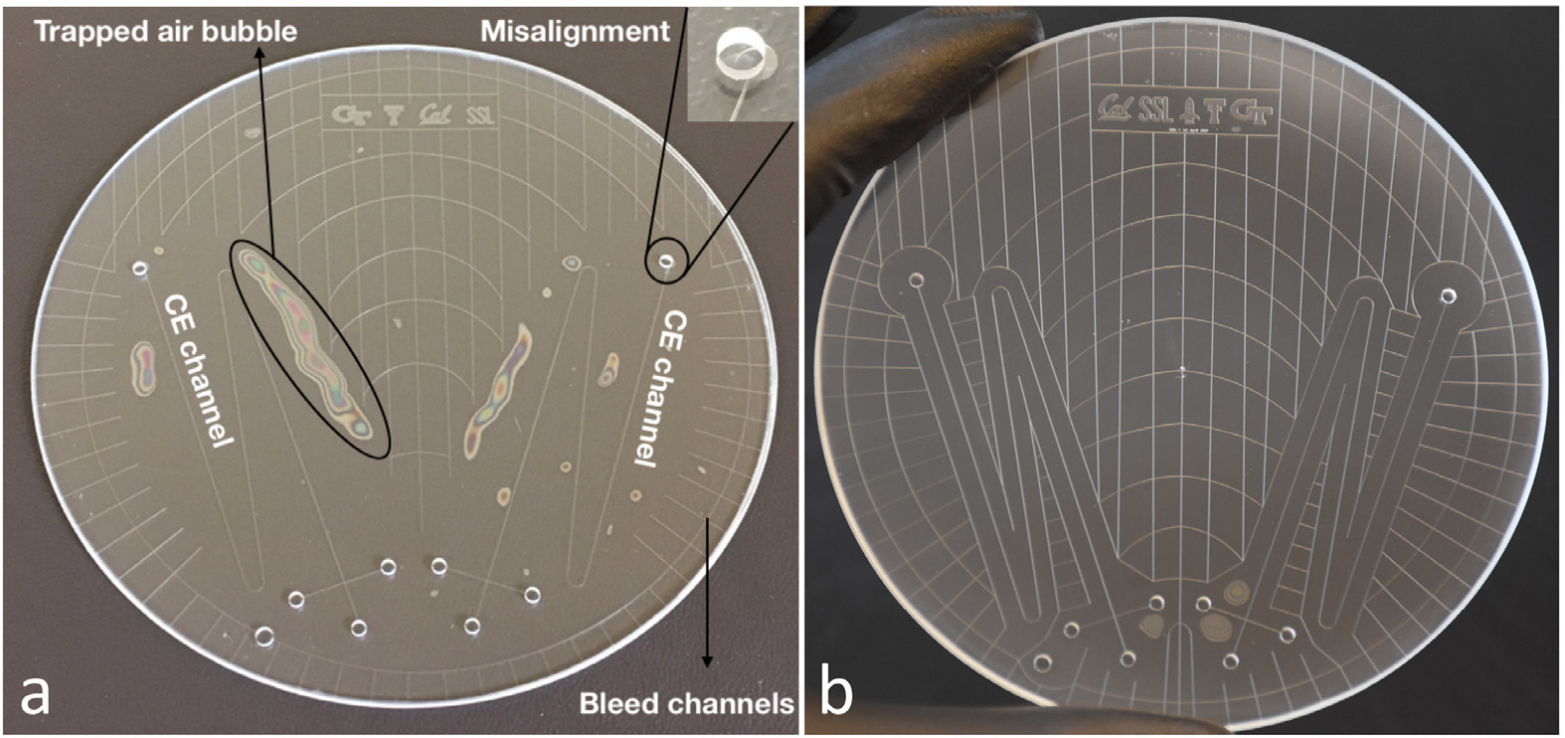
(a) Image of a poor-quality bonded CE chip with multiple non-bonded areas and misaligned CE reservoir holes; (b) Image of a higher quality bonded chip through an improved mask design with respect to bleed channels. Bonding imperfections in this case are mostly due to the roughness of the drilled holes.

A list of tips is included below in order to provide a troubleshooting guide for the most common failures during the glass bonding process displayed in Fig. 8. Additional tips to overcome common failures during the glass bonding process can be found here [29].

1. Misaligned CE reservoir holes:
  • If Macor blocks, especially the bottom one, is not completely flat, the wafers might slightly slide on top of each other during the assembly and bonding processes. This will cause misalignment issues and as can be seen in Fig. 8a; top drilled holes are not aligned with the etched CE holes. To prevent this, make sure ceramic blocks are flat and resurfaced frequently.
2. Poor bonding quality with non-bonded areas (Fig. 8):
  • This may have been caused by insufficient cleaning of the glass wafers prior to bonding. Ensure all steps described in the Standard Cleaning Procedure are done correctly. The presence of the air bubbles in the bonding interface minimizes the adhesion strength and can reduce the separation efficiency if a void happens to extend into the CE channels.
  • If the wafers are clean and flat and there are still air pockets being trapped in the bonding interface, the mask has to be redesigned to include bleed channels. The quality of the bonding can be significantly improved by uniformly placing bleed channels across the entire mask and reasonably close to the CE channels. These channels allow the trapped air in the bonding interface to escape during the bonding process. As can be seen in Fig. 8a, the bleed channels need to be extended towards the CE channels where air pockets are being trapped. Fig. 8b illustrates a higher quality bonded chip as a result of an improved mask design with respect to exhaust lines. However, there are still a few air bubbles present near the input CE reservoir holes. These bubbles come from the roughness of the drilled holes. This can be prevented by frequently exchanging the drill bits with a fresh one during the drilling process as well as extending the exhaust lines to the close vicinity of the holes.

**Fig. 9:**
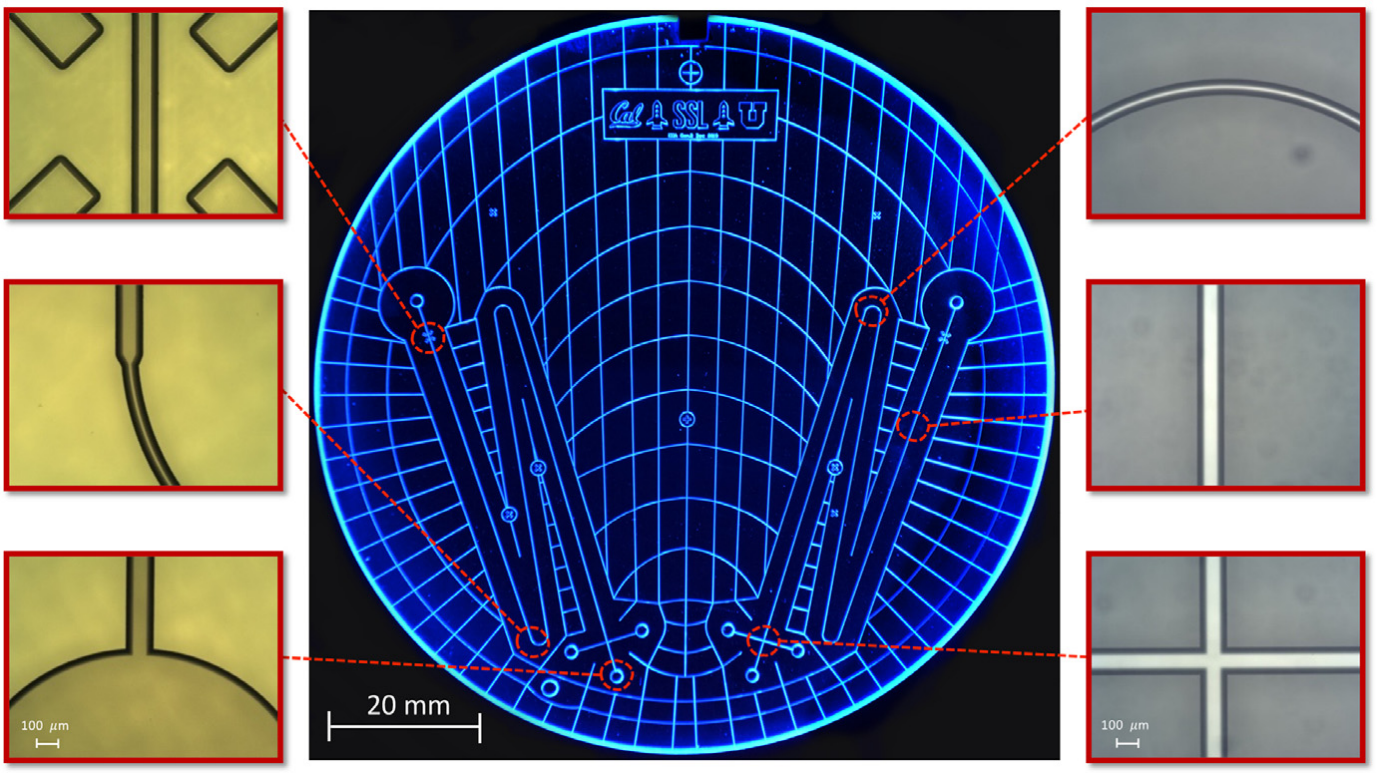
Image of bonded chip produced at Berkeley Microfabrication Facility. Bonding temperature: 668°C for 6 h. All microchannels are defect free, the drilled holes are in perfect alignment with the etched holes, and no trapped air bubbles were produced during bonding process making this a fully functional wafer. The optical images demonstrate the features in borofloat glass etched to a depth of 30 µm using 49% HF and amorphous silicon as the etch mask.

Fig. 9 presents images of a high-quality fabricated CE chip produced at Berkeley Microfabrication Facility using all the procedures described here. The CE reservoir holes are made with a high-speed drill and are in perfect alignment with the etched holes. Bleed channel design is optimized to prevent the formation of air bubbles in the bonding interface. Finally, all etched features are defect free and have been etched to a depth of 30 µm using 49% HF into the borofloat. It is worth mentioning that the success rate of the bonding process using all the techniques described above is 100% and all finished devices were defect free and maintained excellent bonding quality.

#### Performance validation

The performance of our fabricated devices was validated by performing CE separations of a mixture of fluorescently labeled amino acids [18]. A stock solution of Pacific Blue succinimidyl ester (ex. 405 nm, em. 425 nm) was used to label amino acid solutions in 30 mM borate buffer, pH 8.5. An equimolar mixture of ten amino acids including Histidine (His), Leucine (Leu), Valine (Val), Serine (Ser), Alanine (Ala), Glycine (Gly), Glutamic Acid (Glu), Aspartic Acid (Asp), alpha-aminoisobutyric (AIB), gamma-aminobutyric (GABA) were mixed with a 5x molar excess of Pacific Blue succinimidyl ester and allowed to react overnight. Separations were performed with a custom CE system and detected with an epi-fluorescence microscope equipped with a 405 nm diode laser for excitation, a 425 nm long-pass emission filter, a 32x/0.4 objective, photomultiplier tube (PMT) detection, and a fast-switching high voltage power supply (HVPS). Switching control for the HVPS and data acquisition were achieved through a LabView program.

**Fig. 10:**
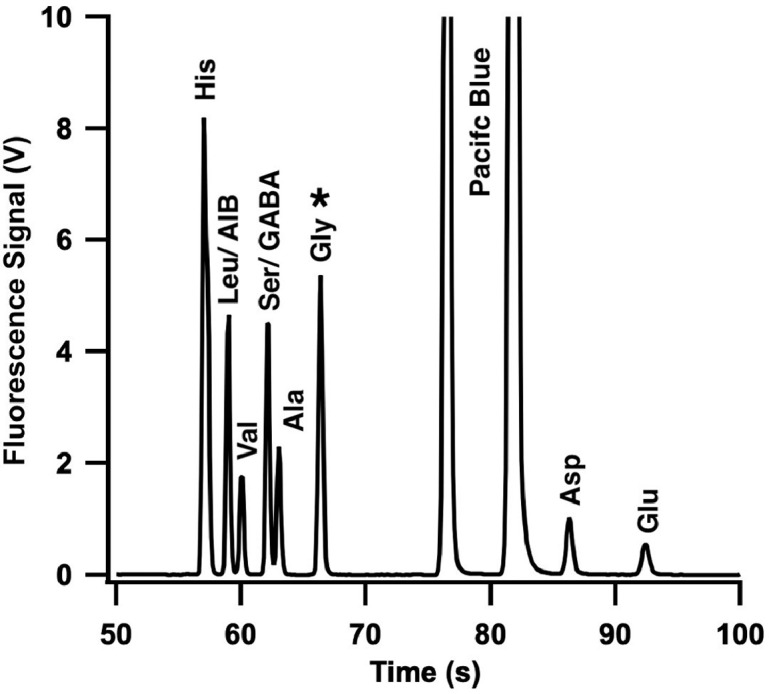
Separation of a mixture of amino acids using a CE chip fabricated at the Berkeley Microfabrication Facility. Electropherogram of ten Pacific Blue labeled amino acids in 30 mM borate buffer with pH 8.5 and an applied field of 670 V/cm between the injection and detection points. The two hydrolysis products of PB are also indicated.

The sample was injected into the CE channel using an electrokinetic cross-t injection with an applied field of 670 V/cm across the sample-to-waste channel for 60 s. CE was carried out with a field 670 V/cm in the separation channel. Fig. 10 presents a representative electropherogram of the separation of ten PB-labeled amino acids completed in less than 100 s. Peak metrics reveal baseline resolution for 8 amino acids and the two hydrolysis products from Pacific Blue dye with average peak efficiencies of 140,000 plates and excellent migration time reproducibility (0.3% RSD) indicative of high quality CE channel fabrication. Two of the amino acids Leu/AIB and Ser/GABA were found to co-migrate under the conditions used. This validation demonstrates the quality of CE separations that can be achieved in a routine fashion when device preparation follows the protocols presented above.
